# Improving the production of AHL lactonase AiiO-AIO6 from *Ochrobactrum* sp. M231 in intracellular protease-deficient *Bacillus subtilis*

**DOI:** 10.1186/s13568-020-01075-7

**Published:** 2020-08-05

**Authors:** Rui Xia, Yalin Yang, Xingliang Pan, Chenchen Gao, Yuanyuan Yao, Xuewei Liu, Tsegay Teame, Fengli Zhang, Juan Hu, Chao Ran, Zhen Zhang, Jihong Liu-Clarke, Zhigang Zhou

**Affiliations:** 1 Sino-Norwegian Fish Gastrointestinal Microbiota Joint Lab, Feed Research Institute, Chinese Academy of Agricultural Sciences, Beijing, 100081 China; 2 Key Laboratory for Feed Biotechnology of the Ministry of Agriculture, Feed Research Institute, Chinese Academy of Agricultural Sciences, Beijing, 100081 China; 3 NIBIO, Norwegian Institute of Bioeconomy Research, 1431 Ås, Norway

**Keywords:** AHL lactonase, *Bacillus subtilis*, Quorum quenching, Intracellular protease-deletion mutant

## Abstract

Quorum quenching (QQ) blocks bacterial cell-to-cell communication (i.e., quorum sensing), and is a promising antipathogenic strategy to control bacterial infection via inhibition of virulence factor expression and biofilm formation. QQ enzyme AiiO-AIO6 from *Ochrobactrum* sp. M231 has several excellent properties and shows biotherapeutic potential against important bacterial pathogens of aquatic species. AiiO-AIO6 can be secretory expressed in *Bacillus subtilis* via a non-classical secretion pathway. To improve AiiO-AIO6 production, four intracellular protease-deletion mutants of *B. subtilis* 1A751 were constructed by individually knocking out the intracellular protease-encoding genes (*tepA, ymfH, yrrN* and *ywpE*). The AiiO-AIO6 expression plasmid pWB-AIO6BS was transformed into the *B. subtilis* 1A751 and its four intracellular protease-deletion derivatives. Results showed that all recombinant intracellular protease-deletion derivatives (BSΔ*tepA*, BSΔ*ymfH*, BSΔ*yrrN* and BSΔ*ywpE*) had a positive impact on AiiO-AIO6 production. The highest amount of AiiO-AIO6 extracellular production of BSΔ*ywpE* in shake flask reached 1416.47 U/mL/OD_600_, which was about 121% higher than that of the wild-type strain. Furthermore, LC–MS/MS analysis of the degrading products of 3-oxo-C8-HSL by purification of AiiO-AIO6 indicated that AiiO-AIO6 was an AHL-lactonase which hydrolyzes the lactone ring of AHLs. Phylogenetic analysis showed that AiiO-AIO6 was classified as a member of the α/β hydrolase family with a conserved “nucleophile-acid-histidine” catalytic triad. In summary, this study showed that intracellular proteases were responsible for the reduced yields of heterologous proteins and provided an efficient strategy to enhance the extracellular production of AHL lactonase AiiO-AIO6.

## Introduction

Quorum quenching (QQ) blocks bacterial cell-to-cell communication (i.e., quorum sensing), and is a promising antipathogenic strategy to control bacterial infection via inhibition of virulence factor expression and biofilm formation. QQ strategy has been applied to many fields such as aquaculture, crop production and anti-biofouling (Grandclément et al. 2016). QQ enzyme AiiO-AIO6 from *Ochrobactrum* sp. M231 has excellent properties such as high ion and chemical resistance, high thermostability, broad-spectrum substrate specificity and high enzyme activity, and shows biotherapeutic potential against important bacterial pathogens of aquatic organisms (Zhang et al. 2011). AiiO-AIO6 can be secretory expressed in *Bacillus subtilis* via a non-classical secretion pathway (Pan et al. 2016). However, the secretion level of AiiO-AIO6 in *B. subtilis* is low and needs to be improved.

Host proteases have been considered as one of the major factors limiting the production of heterologous proteins in *B. subtilis*. Many studies have showed that the deletions of protease genes have improved the yields of many recombinant proteins, such as the use of protease-deficient strains to enhance extracellular pullulanase production in *B. subtilis* (Zhang et al. 2018). However, these studies have focused on knocking out membrane-bound, cell wall-associated or secreted protease genes; few studies have involved the deletion of intracellular proteases. *B. subtilis* encodes three proteases (HtrA, HtrB and WprA) that are known to be functional at the wall/membrane interface or in the wall itself (quality control proteases), and seven proteases (AprE, Bpr, Epr, Mpr, NprB, NprE and Vpr) that are secreted into the culture medium (feeding proteases). Previous work has shown that some or all of these proteases were responsible for the reduced yields of various heterologous proteins (Westers et al. 2008; Wu et al. 1993, 2002). Intracellular proteases also play an important role in quality control and act as a major barrier to the production of certain secreted recombinant proteins (Molière and Turgay 2009; Park and Schumann 2015; Westers et al. 2004b). For example, an intracellular protease such as AprX was involved in degradation of a heterologous protein during the late stationary growth phase and the AprX mutant exhibited enhanced production of heterologous proteins (Kodama et al. 2007).

The aim of this study was to compare and evaluate the effect of these intracellular proteases such as serine protease (TepA), cysteine protease (YwpE), metalloproteinase (YmfH) and unknown protease (YrrN), on the secretion of AiiO-AIO6 by *B. subtilis*. The degradation mechanism and homology of AiiO-AIO6 was also analyzed by LC–MS/MS and phylogenetic tree.

## Materials and methods

### Bacterial strains, plasmids and growth conditions

The bacterial strains and plasmids used in this study are described in Additional file 1: Tables S1 and S2. All mutated *B. subtilis* strains were derivatives of *B. subtilis* strain 1A751. All *B. subtilis* strains were grown in super-rich medium containing 25 g Bacto tryptose, 20 g Bacto yeast extract and 3 g K_2_HPO_4_ per liter (pH 7.5) or agar plates with ampicillin (100 μg/mL), spectinomycin (100 μg/mL), zeocin (25 μg/mL) and kanamycin (25 μg/mL).

### Construction of intracellular protease deletion mutants

The primers used in this study are summarized in Additional file 1: Table S3. To create the gene deletion loci for *tepA, ymfH, yrrN* and *ywpE*, the genes and flanking regions (about 2000 bp) were amplified from chromosomal DNA using their respective upstream and downstream primers. These PCR products were ligated into T-vectors to generate template vectors pT-tepA, pT-ymfH, pT-yrrN, and pT-ywpE, which were then used as templates to amplify the 5′ and 3′ flanks of these genes. pPIC9K was used as template to amplify the zeocin resistance gene. The zeocin resistance gene replaced the deletion gene and was inserted between the 5′ flanks of the deletion gene and the 3′ flanks of the deletion gene of template vectors to generate antibiotic selection marker knockout vectors pΔ*tepA*, pΔ*ymfH*, pΔ*yrrN* and pΔ*ywpE.* Knockout vectors were transformed to *B. subtilis* 1A751. The suspect mutant cells resistant to zeocin were further identified by diagnostic PCR with the upstream forward primer/the downstream reverse primer of these deletion genes and the upstream forward primer of 5′ flanks of these deletion genes/the downstream reverse primer of zeocin gene. The mutant was further confirmed by DNA sequencing.

### Secretory expression of AiiO-AIO6

The AiiO-AIO6 expression plasmid pWB-AIO6BS was constructed following protocols as described previously (Pan et al. 2016). pWB-AIO6BS was transformed into the *B. subtilis* 1A751 and its four intracellular protease gene deletion derivatives. The secretion of AiiO-AIO6 from *B. subtilis* was studied using pWB-AIO6BS-harboring strains 1A751, BSΔ*tepA*, BSΔ*ymfH*, BSΔ*yrrN,* and BSΔ*ywpE*. *B. subtilis* cells were cultured in SR medium with kanamycin (25 μg/mL) at 200 rpm for 24 h at 30 °C. Bacterial growth was monitored by measuring optical density at 600 nm with the BioPhotometer plus of Eppendorf AG (Hamburg, Germany). Culture supernatant was separated from *B. subtilis* culture by centrifugation at 12,000*g* (10 min, 4 °C) and subjected to AHL-lactonase activity bioassay. Proteins in the supernatants were precipitated with two volume of ice-cold acetone, and then acetone precipitations were separated on 12% polyacrylamide (TGX Stain-Free FastCast Acrylamide Kit, Bio-Rad) and transferred to polyvinylidene difluoride (PVDF) membranes (Immobilon; 0.45 μm pore size; Millipore). All stain-free gels were imaged with the Gel Doc XR+ documentation system (Bio-Rad). Western blot analysis was carried out using monoclonal mouse-anti-His-Tag antibody (TianGen, China) as the primary antibody and performed using One Step Western Kit HRP (mouse) according to the manufacturer’s instructions (CW Biotech Company Beijing, China).

### Enzyme assays

One unit of AHL lactonase activity was defined as the amount of enzyme that hydrolyzed 1 nmol 3-oxo-C_8_-HSL per minute. For the hydrolysis assay, the reaction mixture (500 μL) contained 50 μL AiiO-AIO6, 0.248 mM 3-oxo-C8-HSL, and 10 mM PBS (pH 7.0). The reaction was terminated at 70 °C for 10 min after the mixture was incubated at 30 °C for 30 min. The reaction mixtures were centrifuged at 10,000*g* for 10 min at 4 °C and the supernatants after 0.22 μm filtration were used for HPLC analysis and quantification. The residual 3-oxo-C8-HSL was separated in a Shimadzu InertSustain C18 column at 30 °C with a constant flow rate of 1 mL/min in isocratic elution with aqueous–organic mobile phase containing 0.375% triethylamine/acetonitrile (64:36, V/V), and then detected with an UV/visible light detector (waters) at 201 nm. The remaining AHLs were quantified by calculating the peak areas for a given retention time compared to AHL solutions of known concentrations. Furthermore, for the control, AiiO-AIO6 was replaced with inactivated AiiO-AIO6 by heat treatment (100 °C for 5 min). All determinations were performed in four replicates.

An enzyme assay was carried out using assay systems that consisted of mixtures of PBS (pH 7.0) and 0.249 to 0.870 nM 3-oxo-C8-HSL and an incubation time of 30 min at 30 °C, and the corresponding reaction rate was calculated. The Michaelis–Menten equation in GraphPad Prism 8 was used to measure the Vmax, Km and Kcat of Michaelis–Menten kinetics.

### LC–MS/MS analysis of the hydrolysis products of AHL by AiiO-AIO6

The above hydrolysis products of AHL by purified AiiO-AIO6 were extracted three times with ethyl acetate. The combined organic phase was then evaporated to dryness. The samples were dissolved in methanol and separated by a 50-min isocratic elution (mobile phase: methanol: water (60:40; v/v); flow rate: 0.25 mL/min) on a Thermo Scientific Dionex Ultimate 3000 UPLC system with C18 column. MS experiments were conducted on a Thermo Q Exactive mass spectrometer (Thermo Fisher Scientific, San Jose, CA, USA) in a data-dependent acquisition mode with mass range 50 to 750 m/z using the Xcalibur 2.1.2 software followed by ten data-dependent MS/MS scans. The full scan and fragment spectra were collected with resolutions of 70,000 and 17,500 respectively.

### Phylogenetic analysis

For AHL lactonase AiiO-AIO6, homologous protein sequences were retrieved from NCBI after identification by BLAST. Multiple sequence alignment was constructed using DNAMAN. The phylogenetic tree was generated by the neighbor-joining method with the ClustalW (MEGA7).

## Results

### Identification of intracellular protease-deficient *B. subtilis* strains

**Fig. 1:**
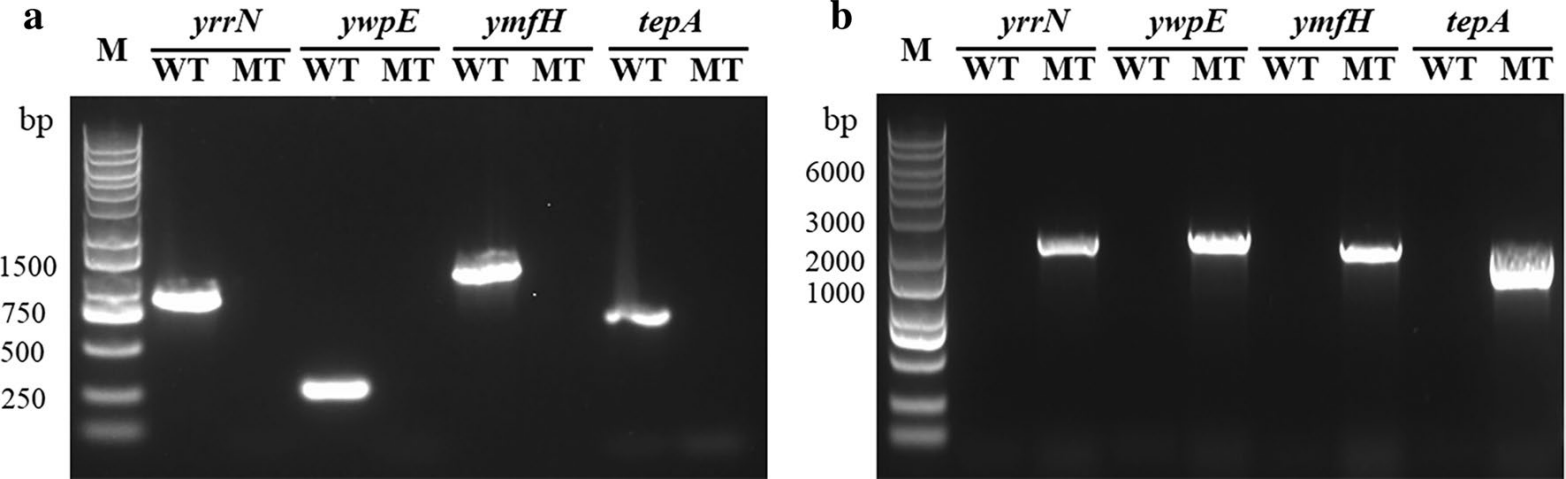
Confirmation of the disruptions of *yrrN*, *ywpE*, *ymfH* and *tepA*. PCR products were amplified from the genomes of wild‐type (lane WT) and mutant (lane MT) strains using verification primer pairs as described in “Materials and methods” section. Lane M: DNA marker (M115, Genestar)

In order to study the effects of intracellular proteases on AiiO-AIO6 secretion expression, four intracellular proteases, TepA, YmfH, YrrN and YwpE, were deleted from the genome of *B. subtilis* 1A751, using the knockout vectors pΔ*tepA*, pΔ*ymfH*, pΔ*yrrN*, and pΔ*ywpE*, respectively. First, specific primers of knockout genes were used, no products were amplified in mutant strains which confirmed the successful knocked out. Then the resulting *B. subtilis* knockout strains (BSΔ*tepA*, BSΔ*ymfH*, BSΔ*yrrN* and *BS*Δ*ywpE*) were confirmed by PCR with the upstream forward primer of 5′ flanks of these deletion genes and the downstream reverse primer of zeocin resistance genes. No product was amplified from the wild-type strain, while PCR products with expected sizes were amplified from intracellular protease-deficient *B. subtilis* strains using their respective primer pairs (Fig. 1b).

### Secretory production of AiiO-AIO6 in intracellular protease-deficient *B. subtilis* strains and its kinetic characterization

**Fig. 2:**
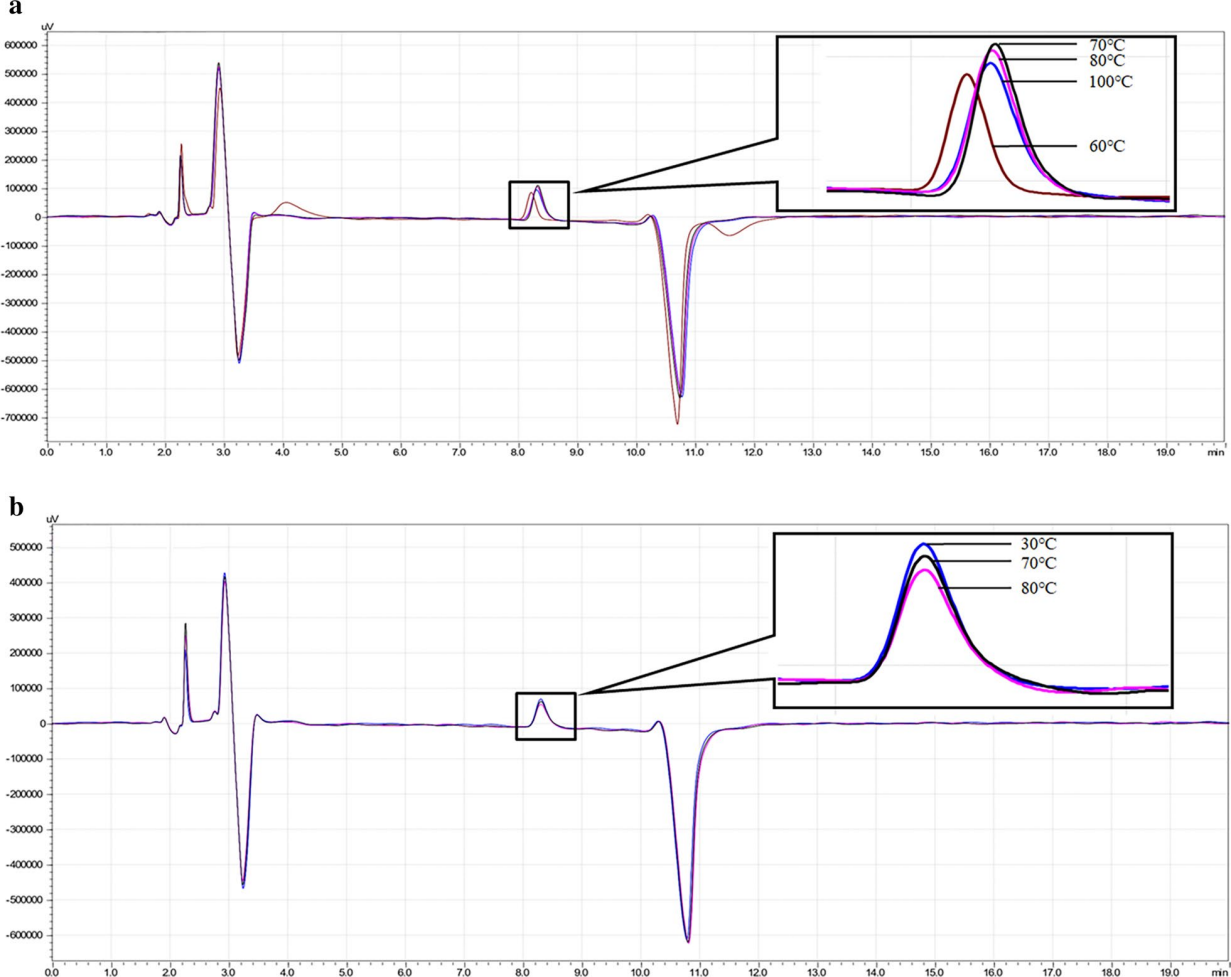
The inactivation of AiiO-AIO6 and the stability of 3-oxo-C8-HSL under different temperatures. **a** AiiO-AIO6 was heated at 60, 70, 80, and 100 °C (as control with 100% inactivation rate) for 10 min and then reacted with 3-oxo-C8-HSL at 30 °C for 20 min. The reaction products were analyzed by HPLC to quantify 3-oxo-C8-HSL. **b** 3-oxo-C8-HSL solution was treated at 30 (as control with 0% lactonolysis rate), 70 and 80 °C for 10 min then detected directly by HPLC to quantify 3-oxo-C8-HSL

Studies showed that the acidic group of SDS binds to the reversed-phase column where it serves as an ion exchanger and can interfere with RPLC. To avoid the interference of SDS in the separation of AHL by HPLC, we inactivated enzyme reaction by heat treatment. To determine the optimal temperature at which AiiO-AIO6 was inactive and 3-oxo-C8-HSL was still stable, thermal inactivation of AiiO-AIO6 was measured. The inactivation rate of AiiO-AIO6 was 66.50%, 99.20%, 99.97%, 100% (Fig. 2a) after heating at 60, 70, 80, and 100 °C for 10 min, respectively. Then AHL after treatment at 30, 70 and 80 °C for 10 min was detected by HPLC and the remaining amount of AHL were calculated as 100%, 96.49% and 85.62% (Fig. 2b), respectively. Therefore, the optimal inactive temperature for enzyme reaction was 70 °C (Additional file 1: Tables S4, S5).

**Fig. 3:**
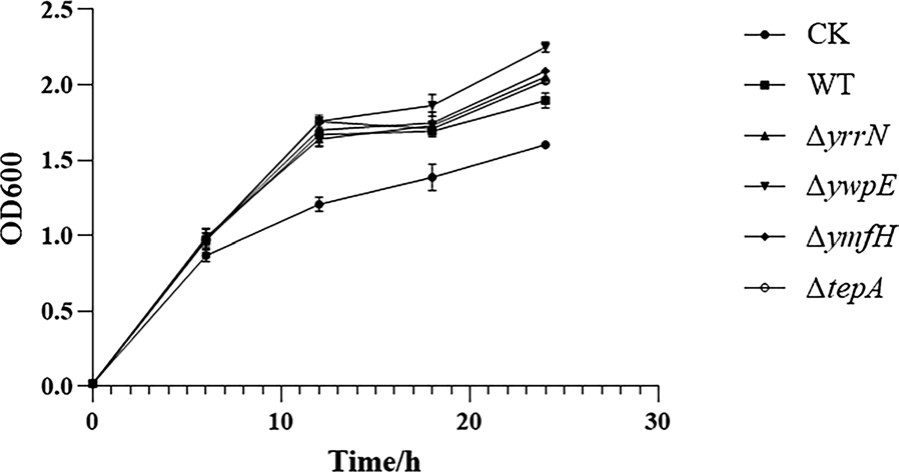
Growth curve of intracellular protease-deficient strains. CK, pWB980/BS1A751, wild-type (WT), pWB980-AIO6BS/BS1A751; ∆*ymfH*,∆*yrrN*, ∆*ywpE*, ∆*tepA*: intracellular protease-deficient mutants harboring the expression vector pWB980-AIO6BS

**Fig. 4:**
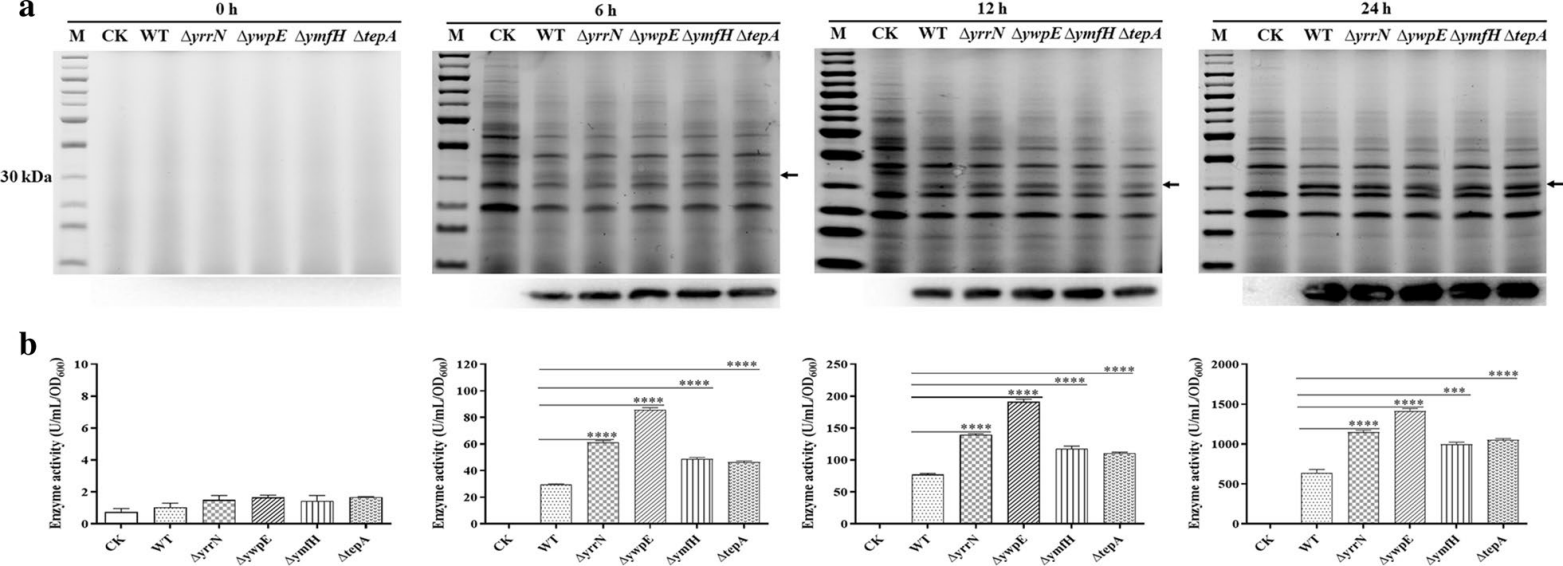
Secretion of AiiO-AIO6 by intracellular protease-deficient strains. **a** SDS-PAGE (up) and Western blotting (down) of AiiO-AIO6 secreted in culture medium derived from wild-type (WT) strain 1A751 or intracellular protease-deficient mutants after different time points cultivation at 37 °C. M, protein marker; CK, pWB980/BS1A751, wild-type (WT), pWB980-AIO6BS/BS1A751; ∆*ymfH*, ∆*yrrN*, ∆*ywpE*, ∆*tepA*: intracellular protease-deficient mutants harboring the expression vector pWB980-AIO6BS. **b** Secreted AiiO-AIO6 activity present in wild-type or intracellular protease-deficient mutant culture medium. *p* values were calculated using unpaired two-tailed T-tests. An asterisk indicates a statistically significant difference between two activity values (*p *< 0.05)

By detecting the growth of bacteria, we found that these mutations did not affect bacterial growth of strain 1A751 which compared with the wild-type (pWB980-AIO6BS/BS1A751) (Fig. 3). The culture supernatants from the wild-type strain and mutant strains were analyzed by SDS-PAGE and Western blotting. SDS-PAGE clearly showed that the band intensities of AiiO-AIO6 from the recombinant intracellular protease-deficient strains were significantly higher than that observed in the wild-type *B. subtilis* 1A751 strain (Fig. 4a). Moreover, the AiiO-AIO6 activities in culture supernatants of recombinant mutant strains were significantly higher than that of the recombinant wild-type strain. Recombinant strain BSΔ*ywpE*/pWB-AIO6BS achieved the highest secretion of 1416.47 U/mL/OD_600_ at 24 h, which was about 121% higher than that of the recombinant wild-type strain (Fig. 4b). Under optimal conditions (pH 7.0, 30 °C, 3-oxo-C8-HSL as substrate), the specific activity of purified AiiO-AIO6 was 4.41 × 10^3^ U/mg. Kinetic parameters including Km, Vmax, and Kcat values of AiiO-AIO6 were calculated as 0.4151 mmol/L, 16,949 nmol/mg/min, and 9.34 S^−1^, respectively.

### AIO6 is an AHL lactonase

**Fig. 5:**
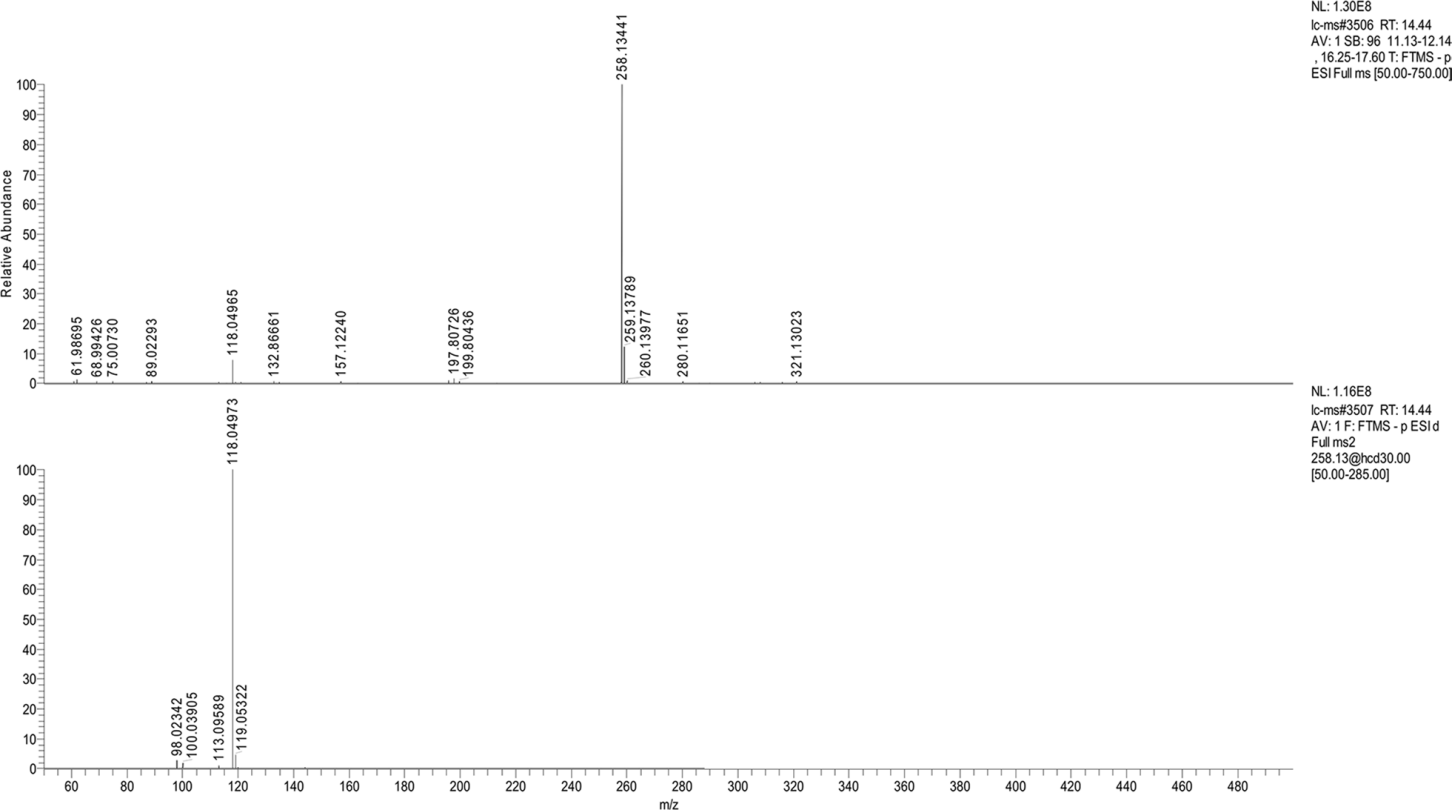
LC–MS/MS analysis of the hydrolysis product of 3-oxo-C8-HSL by AiiO-AIO6. MS^1^ analysis of the 14.44 min HPLC fragment of enzymatic hydrolysates showed a mainprecursor (M−H) ion at m/z of 258.1 (upper panel). MS^2^ spectra of the precursor ion at m/z of 258.1 by tandem mass spectrometry showed a main fragment ion at m/z 118.1 (bottom panel)

To identify which QQ enzyme AiiO-AIO6 belongs to, the degradation products of 3-oxo-C8-HSL by purified AiiO-AIO6 were analyzed using LC–MS/MS. As shown in Fig. 5, LC–MS/MS analysis of the 14.44 min HPLC fraction showed a M−H ion at a mass-to-charge ratio (*m/z*) of 258.1, which was identical to that of the open-ring 3-oxo-C8-HSL, and tandem MS of the precursor ion at a *m/z* of 258.1 showed a characteristic fragment at a *m/z* of 118.0, corresponding to homoserine, resulting from the lactone-opened *N*-(3-oxooctanoyl)-l-homoserine. These results strongly suggested that AiiO-AIO6 was an AHL-lactonase which hydrolyzes the lactone ring of AHLs.

**Fig. 6:**
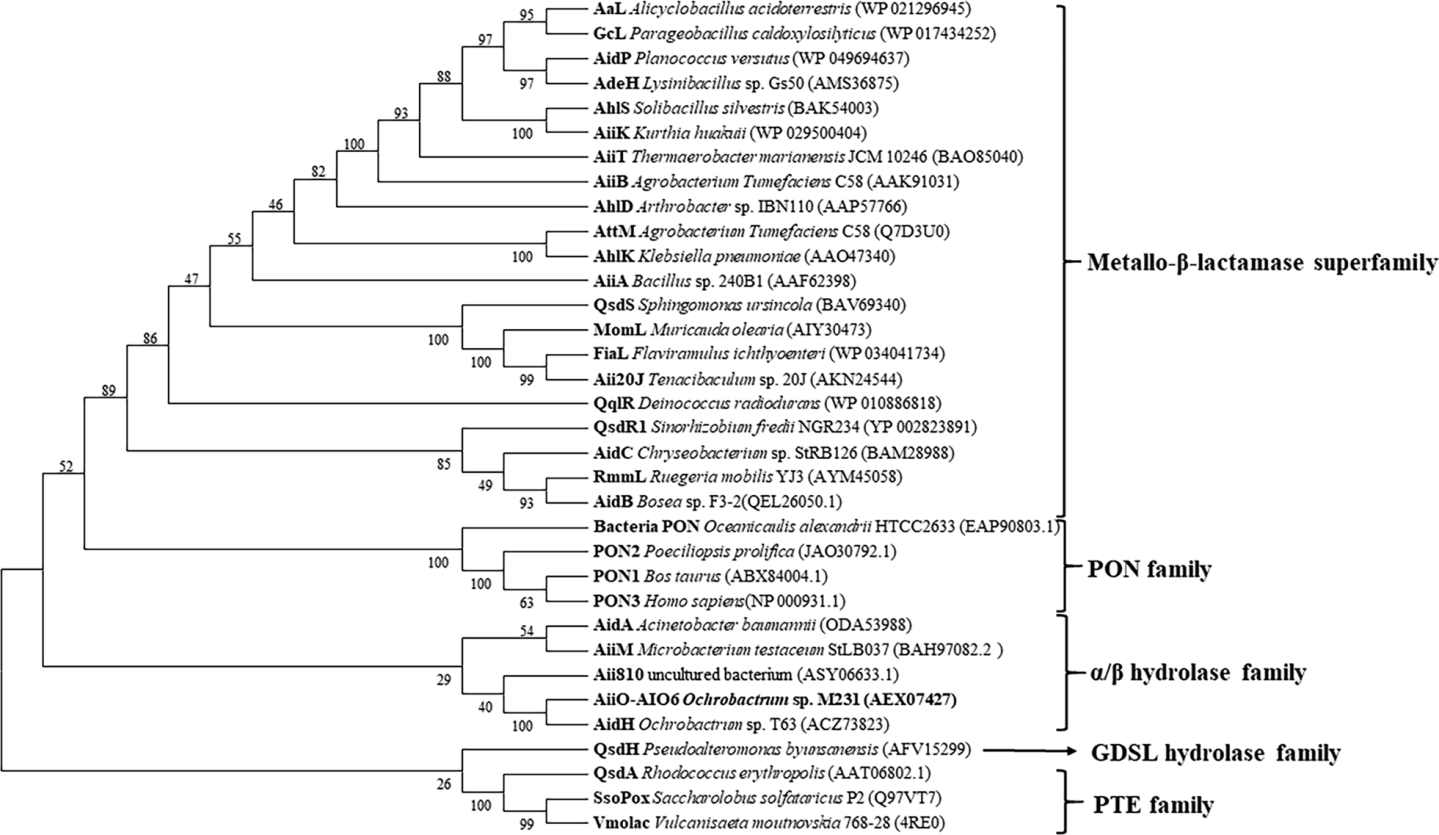
Phylogenetic tree of AiiO-AIO6 and the known lactonases. The phylogenetic analysis was constructed by the neighbor-joining method with the ClustalW (MEGA7). Bootstrap values from 1000 replicates are given on the nodes

**Fig. 7:**
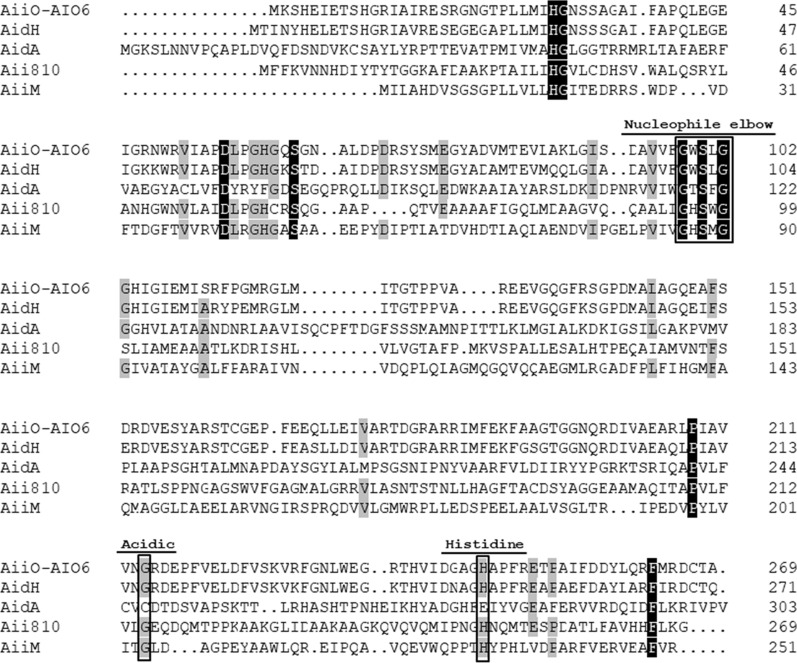
Comparison of amino acid sequences of AiiO-AIO6 and several alpha/beta-hydrolases. The alignment was generated by DNAMAN. Identities are highlighted in white with a black background, and similarities are shaded gray. The catalytic triad residues are boxed with rectangles. The amino acid residues essential for AHL-degrading activity are indicated by asterisks. AiiO-AIO6, AiiO-AIO6 from *Ochrobactrum* sp. M231 (GenBank accession no. AEX07427); AidH, AidH from *Ochrobactrum* sp. T63 (GenBank accession no. GQ849010); AidA, AidA from *Acinetobacter baumannii* (GenBank accession no. ODA53988); Aii810, Aii810 from uncultured bacterium (GenBank accession no. ASY06633.1); AiiM, AiiM from *Microbacterium testaceum* StLB037 (GenBank accession no. BAJ75775)

Phylogenetic analysis of known AHL-lactonase indicated that AiiO-AIO6 was classified as an α/β hydrolase family member (Fig. 6). AiiO-AIO6 shared 84.8%, 13.8%, 12.7% and 10.8% amino acid sequence identity with AidH from *Ochrobactrum* sp. Strain T63 (Mei et al. 2010), Aii810 from metagenome (Kawasaki and Suzuki 1990), AiiM from *M. testaceum* StLB037 (Wang et al. 2010) and AidA from *Acinetobacter baumannii* (López et al. 2017), respectively. Like AidH, AiiO-AIO6 also contained the “nucleophile-acid-histidine” catalytic triad (Ser100, His246 and Glu214) that was conserved among members of the alpha/beta-hydrolase family and was metal-independent AHL-lactonase (Gao et al. 2013) (Fig. 7).

## Discussion

*Bacillus subtilis* has a strong ability to secrete natural or homologous proteins. About 60% of commercially available proteins are produced by *Bacillus*, but the expression types and products of heterogenous proteins are still limited (Fu et al. 2007). Two alternative strategies to improve heterologous protein secretion production are to screen functional signal peptides (Kang et al. 2014) and to delete host proteases to reduce proteolytic degradation (Westers et al. 2004b). Since AiiO-AIO6 was a protein secreted through a non-classical pathway, a conventional signal peptide can not improve its secretion (Pan et al. 2016).

Many studies have focused on engineering of extracellular proteases to reduce extracellular proteolysis. There are few reports on whether deletion of intracellular protease could improve the secretion production of heterogenous protein, so we have tried to use an intracellular protease-deletion strain to improve its secretion. In this study, four intracellular proteases were deleted in order to study their effect on the secretion of AiiO-AIO6. YwpE is one of two putative sortase homologues of *B. subtilis*. YwpE encodes a protein of 102 amino acids with the LxTC motif for the sortase activity at the C-terminus, but lack of a transmembrane anchor at its N-terminus. The study of the role of YwpE in displaying two potential sortase substrates YhcR and YfkN on the cell wall showed that YwpE seems not to play a major role, if any, as a sortase (Nguyen et al. 2011). The peptidase U32 protein YrrN is one member of the yrrMNO operon which is important for the biosynthesis of 5-hydroxyuridine, YrrN is involved in the synthesis of 5-methoxyuridine (Nguyen et al. 2011). The cytoplasmic ClpP-like germination protease TepA is involved in spore outgrowth in *B. subtilis*, and is not proposed to be a signal peptide peptidase but to degrade a specialized family of small DNA-binding proteins during the process of spore outgrowth (Traag et al. 2013; Westers et al. 2004a). YmfH is identified by the presence of Peptidase_M16 and Peptidase_M16_C domains, and is predicted to encode uncharacterized zinc protease with unknown specificity (Hummels et al. 2017). In this study, the deletion of any of the four proteases improved the secretion of AiiO-AIO6, but the degree of improvement was different. Their effect on the secretion of AiiO-AIO6 in *B. subtilis* was YwpE > YrrN > TepA or YmfH, which may be due to the difference in the degradation ability of these proteases to AiiO-AIO6 or the degradation ability of the proteins assisting the secretion of AiiO-AIO6. This study showed that deletion of intracellular protease is also a good strategy to improve the secretion and expression of heterogenous protein.

A simple and accurate method for monitoring enzymatic activity is of fundamental importance to the study of QQ enzymes. At present, the main detection methods for QQ enzyme activity are microbiosensor-based biological detection and chromatography-based physicochemical detection (mainly HPLC) (Liu et al. 2018). Of these, the physicochemical detection method is quantitatively more accurate but requires sample pretreatment. The main method for extracting AHLs from aqueous samples is liquid-to-liquid extraction (Feng et al. 2014; Tan et al. 2014). However, the extractant and extraction steps will affect the extraction efficiency, and there are some problems such as hazardous organic solvent consumption and cumbersome and time-consuming experimental steps. The composition of the enzyme reaction solution is not too complex, so we tried to bypass the extraction step to directly carry out HPLC.

The HPLC method is an efficient method for quantifying AHLs, but QQ enzymes show low sensitivity to most metal ions and chemicals, and AHLs are sensitive to pH and temperature (Yates et al. 2002). Most enzymes such as AiiA (Yates et al. 2002), AiiK (Dong et al. 2018), AidF (Fan et al. 2020) and AiiO-AIO6 can be inhibited by sodium dodecyl sulfate (SDS) (Zhang et al. 2011), so SDS is usually used as an inhibitor to stop the enzyme reaction (Wang et al. 2004). The strongly acidic group of SDS can bind to the reversed-phase column where it will serve as an ion exchanger and can interfere with RPLC separation (Kawasaki and Suzuki 1990), which seriously affects the HPLC detection. Therefore, it is necessary to find an effective method to terminate the enzyme reaction. In this study, we found that 10 min treatment at 70 °C is an effective way to inactivate AiiO-AIO6 and has little effect on the stability of the signal molecule. This method eliminates the extraction process and greatly simplifies the detection of enzyme activity by HPLC. The extraction-eliminated HPLC improved the detection efficiency of QQ enzyme activity.

AiiO-AIO6 and AidH are both members of the α/β hydrolase superfamily, and they do not contain a metal binding motif. Although there is a high sequence similarity between AiiO-AIO6 and AidH, our previous study found that there are some differences between AiiO-AIO6 and AidH in their characterization. We showed that EDTA and various ions (including Mn ions) did not affect the activity of AiiO-AIO6, and the activity of AiiO-AIO6 did not need metal ions (Zhang et al. 2011), which was also consistent with the metal free binding motif. However, manganese ion was very important for the activity of AidH (Mei et al. 2010). Both AiiO-AIO6 and AidH have a broad substrate spectrum; they can both degrade C6-HSL to C10-HSL, 3-oxo-C6-HSL to 3-oxo-C12-HSL and 3-hydroxy-C12-HSL. However, the substrate specificity of AiiO-AIO6 is different from that of AidH. AidH has a broad substrate specificity, and its degradation activity for different substrates showed no significant difference (Gao et al. 2013). However, AiiO-AIO6 has narrow substrate specificity. AiiO-AIO6 exhibited high activity to 3-oxo-C8-HSL but greatly reduced activities to 3-oxo-C6-HSL, C6-HSL, C12-HSL and 3-hydroxy-C12-HSL (Zhang et al. 2011). These differences in their characterization should be due to the difference between their amino acid sequences.

In summary, this study improves the secretion production of AiiO-AIO6 by engineering host intracellular protease, and YwpE protease knockout will be more conducive for secretion of AiiO-AIO6. Termination of enzyme reaction by temperature eliminated the sample extraction, so HPLC without the extraction step showed improved detection efficiency of QQ enzyme activity. AiiO-AIO6 is a lactonase of the α/β hydrolase superfamily. This study provides an alternative strategy for improving the secretion of heterologous protein by *Bacillus*, which will contributes to the study of quenching enzyme properties, and promote the application of AiiO-AIO6 in the disease control of Gram-negative bacteria in aquaculture.

## Supplementary information

**Additional file 1: Table S1.** Bacterial strains. **Table S2.** Plasmids. **Table S3.** Primers. **Table S4.** The remaining amount of AHL under different temperatures. **Table S5.** The inactivation rate of AiiO-AIO6 under different temperatures.

## Data Availability

All the data are presented in the main paper or the additional information.

## Abbreviations

QQ: Quorum quenching
PCR: Polymerase chain reaction
SDS-PAGE: Sodium dodecylsulfate polyacrylamide gel electrophoresis
PBS: Phosphate buffer solution
