# Supplementary material for: Improving the production of AHL lactonase AiiO-AIO6 from Ochrobactrum sp. M231 in intracellular protease-deficient Bacillus subtilis
Source: AMB Express. 2020 Aug 5;10:138. doi: 10.1186/s13568-020-01075-7 (PMC7406587; doi:10.1186/s13568-020-01075-7)
Supplement: Supplementary file 1 — Additional file 1: Table S1. Bacterial strains. Table S2. Plasmids. Table S3. Primers. Table S4. The remaining amount of AHL under different temperatures. Table S5. The inactivation rate of AiiO-AIO6 under different temperatures. [file 13568_2020_1075_MOESM1_ESM.docx]

Additional Information

| **Table S1. Bacterial strains** | | |
| --- | --- | --- |
| **Strain**s **or plasmid**s | **Genotype or characteristics** | **Source or reference** |
| **Strains** |  |  |
| *E. coli* DH5α | F^-^ϕ80*lacZΔM15Δ(lacZYA-argF)*U169 *recA1 endA1 hsd*R17 (rK−, mK+) *phoA supE44 λ-thi-1 gyr*A96 *rel*A1 | Invitrogen, Carlsbad, CA |
| ***B. subtilis*** |  |  |
| *B. subtilis* 1A751 | *his nprR2 nprE18 DaprA3 DeglS102 DbglT bglSRV* | (Wolf et al. 1995) |
| BS(pWB) | 1A751 containing pWB; Km^r^ | This work |
| BS(pWB-AIO6BS) | 1A751 containing pWB-AIO6BS; Km^r^ | This work |
| BS*∆tepA* | 1A751∆*tepA::zeo* | This work |
| BS*∆ymfH* | 1A751∆*ymfH::zeo* | This work |
| BS*∆yrrN* | 1A751∆*yrrN::zeo* | This work |
| BS*∆ywpE* | 1A751∆*ywpE::zeo* | This work |

| **Table S2. Plasmids** | | |
| --- | --- | --- |
| **Plasmids** | **Genotype and/or relevant characteristic(s)** | **Source** |
| pWB | Km^r^, deleted sacB signal of the vector pWB980 | This work |
| pWB-AIO6BS | Km^r^, AIO6BS, pWB | This work |
| pT-tepA | Km^r^, the gene *tepA* and its flanking sequence, | This work |
| pT-ymfH | Km^r^, the gene *ymfH* and its flanking sequence, | This work |
| pT-yrrN | Km^r^, the gene *yrrN* and its flanking sequence, | This work |
| pT-ywpE | Km^r^, the gene *ywpE* and its flanking sequence, | This work |
| p*∆tepA* | Zeo^r^, *tepA* deletion vector | This work |
| p*∆ymfH* | Zeo^r^, *ymfH* deletion vector | This work |
| p*∆yrrN* | Zeo^r^, *yrrN* deletion vector | This work |
| p*∆ywpE* | Zeo^r^, *ywpE* deletion vector | This work |
|  |  |  |

**Table S3. Primers**

| **Primer name** | **Sequence 5′-3′** |
| --- | --- |
| zeocin-F  zeocin-R | GGTCTGATCGATCTCTGCAGTCGCG  ATTTGTCCTACTCAGGAGAGCGTTC |
| tepA-TF  tepA-TR | CAACCTGCTTAAACCAAAATATTTGA  GCTGCACGTGAGAAAGCAGCAATATG |
| ymfH-TF  ymfH-TR | GACGTGGACAGTAACCAGGTACAATC TTTCAGCCGGGGTAAATTGATTCATC |
| yrrN-TF  yrrN-TR | GTATGAACAAATAAATGACTATATAG CAGCAGCCTCCGCGGTTGGAATCCCG |
| ywpE-TF  ywpE-TR | CCGGGCCTTTTCAATATCCAAATGAG  GCCCCAACATTGCAGGATTTTTTCCT |
| ymfH-T-zeo-F  ymfH -T-zeo-R | GAACGCTCTCCTGAGTAGGACAAATCTGACTGTCTGCAAGGTTGTTCCT  CGCGACTGCAGAGATCGATCAGACCGCTGTTCATATTCGATTGGTTTGATC |
| ywpE-T-zeo-F  ywpE-T-zeo-R | GAACGCTCTCCTGAGTAGGACAAATCCGAAGGGCGCCTCGTTGTGAAAG  CGCGACTGCAGAGATCGATCAGACCCCCGGCGCATTGTTGCCGCCCC |
| tepA -JD-up | CAGCCGGAAGCCAAGGTGAACCATTAGCTG |
| ymfH -JD-up | CAAACGCCGCTTCTCGAGAAAGGGCTCCAG |
| yrrN-JD-up | AGGGCTGACGCCAGGGCCGATTGCCAATGC |
| ywpE-JD-up | GAGACAGAGGTGCCTGCATCCGGAGTGCTG |
| zeocin-JD-down | TTCGTGGACACGACCTCCGACCACTCGGCG |
| tepA-JD-F | catcgtatggaaaacacagaagaag |
| tepA-JD-R | cgtccttcttctttccttgcttcat |
| ymfH-JD-F | ccaatcgaatatgaacagcttcagg |
| ymfH-JD-R | aggaacaaccttgcagacagtcag |
| yrrN-JD-F | aaccgtttccttgaagaagaatccgg |
| yrrN-JD-R | aaccagagctcttagtgacgccga |
| ywpE-JD-F | tgtatggccaaaagaatcaaccaattc |
| ywpE-JD-R | gccgggatcaaaaaatgggtgagg |

Reference in S1

Wolf M, Geczi A, Simon O, Borriss R (1995) Genes encoding xylan and beta-glucan hydrolysing enzymes in *Bacillus subtilis*: characterization, mapping and construction of strains deficient in lichenase, cellulase and xylanase. Microbiology 141 ( Pt 2):281-90

**Table S4. The remaining amount of AHL under different temperatures**

| Group | 30℃（CK） | 70℃ | 80℃ |
| --- | --- | --- | --- |
| Peak area | 1115645 | 1076503 | 955246 |
| Percentage of detected signal molecules | 100% | 96.49% | 85.62% |

**Table S5. The inactivation rate of AiiO-AIO6 under different temperatures**

| Group | 100℃（CK） | 80℃ | 70℃ | 60℃ |
| --- | --- | --- | --- | --- |
| Peak area | 1524182 | 1523750 | 1512023 | 1013598 |
| Percentage of detected signal molecules | 100% | 99.97% | 99.20% | 66.50% |
